# *Streptococcus pyogenes* Pneumonia in Adults: Clinical Presentation and Molecular Characterization of Isolates 2006-2015

**DOI:** 10.1371/journal.pone.0152640

**Published:** 2016-03-30

**Authors:** Esther Tamayo, Milagrosa Montes, Diego Vicente, Emilio Pérez-Trallero

**Affiliations:** 1 Biomedical Research Center Network for Respiratory Diseases (CIBERES), San Sebastián, Spain; 2 Microbiology Department, Hospital Universitario Donostia-Instituto Biodonostia, San Sebastián, Spain; 3 Medicine Faculty, University of the Basque Country, UPV/EHU, San Sebastián, Spain; University of South Dakota, UNITED STATES

## Abstract

**Introduction:**

In the preantibiotic era *Streptococcus pyogenes* was a common cause of severe pneumonia but currently, except for postinfluenza complications, it is not considered a common cause of community-acquired pneumonia in adults.

**Aim and Material and Methods:**

This study aimed to identify current clinical episodes of *S*. *pyogenes* pneumonia, its relationship with influenza virus circulation and the genotypes of the involved isolates during a decade in a Southern European region (Gipuzkoa, northern Spain). Molecular analysis of isolates included *emm*, multilocus-sequence typing, and superantigen profile determination.

**Results:**

Forty episodes were detected (annual incidence 1.1 x 100,000 inhabitants, range 0.29–2.29). Thirty-seven episodes were community-acquired, 21 involved an invasive infection and 10 developed STSS. The associated mortality rate was 20%, with half of the patients dying within 24 hours after admission. Influenza coinfection was confirmed in four patients and suspected in another. The 52.5% of episodes occurred outside the influenza seasonal epidemic. The 67.5% of affected persons were elderly individuals and adults with severe comorbidities, although 13 patients had no comorbidities, 2 of them had a fatal outcome. Eleven clones were identified, the most prevalent being *emm*1/ST28 (43.6%) causing the most severe cases.

**Conclusions:**

*S*. *pyogenes* pneumonia had a continuous presence frequently unrelated to influenza infection, being rapidly fatal even in previously healthy individuals.

## Introduction

The global burden of *Streptococcus pyogenes* disease is high, causing a wide range of mild to severe clinical manifestations that comprise an important cause of morbidity and mortality worldwide. In high income countries, the way that the disease manifests itself has changed during the last few decades. During the mid-20^th^ century, *S*. *pyogenes* was a common cause of epidemic outbreaks and community-acquired pneumonia (CAP) [1–3]. Currently *S*. *pyogenes* is considered a rare cause of community acquired pneumonia, being a clinical entity seen only sporadically after an influenza infection [4–5]. However, the involvement of *S*. *pyogenes* in lower respiratory tract infections is not infrequently seen during the course of invasive infections, which is associated with an exceptionally high mortality rate [6]. Despite of the severity of the illness, few studies have been designed to comprehensively describe large *S*. *pyogenes* pneumonia case series [7,8], with most recent publications being case reports [9–11]. Although some molecular and epidemiological data regarding *S*. *pyogenes* pneumonic episodes can be extracted from studies reporting invasive *S*. *pyogenes* disease, they are not focused on pneumonia and precise data are scarce. The aims of the present study were to describe the clinical features, prognosis and relation with influenza infection of *S*. *pyogenes* pneumonia over a decade and to determine the molecular characteristics (*emm*-type, sequence type, antimicrobial resistance determinants, and superantigen profile) of involved isolates.

## Materials and Methods

### Study area and sample

The study was conducted at Hospital Universitario Donostia, which is the referral hospital of the province of Gipuzkoa, northern Spain, and attends a population of about 350,000 adults >18 years old (annual range 348,726 to 354,475 inhabitants), with an annual mean of 50,640 adult admissions. Medical charts of adult patients with a diagnosis of invasive *S*. *pyogenes* infection or with a *S*. *pyogenes* isolate obtained from lower respiratory tract specimen between January 2006 and December 2015 were revised.

Definition of *S*. *pyogenes* pneumonia was based on the presence of clinical findings (cough, fever, sputum production, and pleuritic chest pain), demonstrable infiltrate on chest radiograph or other imaging techniques and a positive *S*. *pyogenes* culture obtained from blood, pleural fluid, or bronchial secretions (bronchoalveolar lavage, bronchial aspirate, or sputum). Bronchial secretions yielding *S*. *pyogenes* positive cultures were included in the study when it appeared as a single or predominant pathogen. In addition, sputum samples needed to demonstrate > 25 leukocytes and < 10 squamous epithelial cells per low power field on direct Gram-stain.

Pneumonia was considered invasive when *S*. *pyogenes* was isolated from a sterile site, or when obtained from a non-sterile lower respiratory tract site but the clinical presentation and analytical data (Streptococcal Toxic Shock Syndrome (STSS), elevated procalcitonin level, etc.) were consistent with invasive disease.

Isolates were confirmed as *S*. *pyogenes* by routine identification (colony morphology, beta-hemolysis on blood agar plates, agglutination with specific antisera [Slidex, Streptokit; bioMérieux, Marcy l’Etoile, France]), and MALDI-TOF (Matrix Assisted Laser Desorption Ionization-Time of Flight, mass spectrometry analysis Biotyper 3.0, Bruker Daltonics Inc. Billerica, MA, USA).

To relate the streptococcal pneumonic cases with the influenza virus circulation, the influenza rate (epidemic threshold ≥ 80 cases per 100,000 inhabitants) during the previous weeks and coinciding with each *S*. *pyogenes* pneumonia case were recorded (Red Nacional de Vigilancia Epidemiológica. Sistema de Vigilancia de la Gripe en España. ISCIII http://vgripe.isciii.es/gripe/inicio.do). In patients diagnosed with pneumonia and suspicion of flu during the seasonal influenza period, PCR influenza tests (AH1, AH3 and B) were performed.

Demographic and clinical variables were obtained from the patient’s medical charts. Only heavy smokers, those smoking > 20 cigarettes per day, were included among smokers. The Pneumonia Severity Index developed by Fine *et al* [12] was assigned in each patient. Mortality within 30 days was recorded.

### Antimicrobial susceptibility testing

Minimum inhibitory concentrations (MICs) were determined by the broth microdilution method using Sensititre Microtiter Trays (Trek Diagnostics Systems, East Sussex, UK) and cation-adjusted Mueller-Hinton II broth (bioMérieux, Marcy l’Etoile, France) supplemented with 3–5% v/v lysed horse blood. Interpretation was performed according to the Clinical and Laboratory Standards Institute guidelines [13].

### Molecular characterization of isolates

All isolates were characterized by sequencing the 180nt of the 5’ variable region of the *emm* gene (http://www.cdc.gov/streplab/protocol-emm-type.html) and by multilocus sequence typing (http://pubmlst.org/spyogenes/). In isolates showing erythromycin resistance, detection of the macrolide resistance genes *erm*(B), *erm*(A)TR and *mef* was performed as previously described [14]. In isolates with reduced susceptibility to fluoroquinolones (ciprofloxacin MIC ≥2 mg/L) the *parC* and *gyrA* genes were sequenced [15]. Clone was defined by the combination of the *emm*-type, sequence type (ST) and antimicrobial susceptibility pattern.

Detection of *ssa*, *speA*, *speC* and *smeZ* superantigen genes was performed in two multiplex PCR-s using the chromosomally encoded virulence factor genes *speB* and *slo* as successful reaction controls as previously described [16].

### Ethics

The study was an observational, laboratory-based, surveillance study with review of medical records. The patient information was anonymized and study investigators and research associates had no direct patient contact and the study protocol involved no change in patient care or management; all decisions regarding patient investigation and treatment were at the discretion of the attending physician. The institutional ethics committee (Ethics Committee for Clinical Research of the Health Area of Gipuzkoa), specifically approved this study.

### Statistical Analysis

The associations of the variables of sex, *emm*-type, STSS, predisposing conditions, bilobar or multilobar pneumonia, season, and patient evolution (death or survival) were calculated by Fisher’s exact probability test. To analyze the independent effect of each variable in relation to patient outcome, we performed a logistic regression analysis including death and variables with an initial *P* value of ≤ 0.2 tested in the bivariate analysis. Data were analyzed with the IBM SPSS Statistics, Software version 22.

## Results

### Incidence

From January 2006 to December 2015, 40 *S*. *pyogenes* pneumonia episodes were detected. The number per annual period ranged from 1 to 8 episodes, without any significant trend during the study period (annual average incidence 1.14 episodes per 100,000 inhabitants, range 0.29–2.29).

Most cases occurred in winter (n = 26, 65%) and spring (n = 10, 25%). Nineteen cases (47.5%) occurred when the influenza virus circulation reached the epidemic threshold (Table 1) but only five cases (four confirmed and one suspected) had relation with previous or concomitant influenza infection. There were 8 patients whose pneumonia was diagnosed during a flu period in which a PCR influenza test result was not available. Only one of these 8 patients was suspected of having flu, although in this patient the test was not performed or not recorded.

**Table 1:** Description of the 40 *Streptococcus pyogenes* pneumonia episodes in adults and molecular characteristics of involved isolates. Gipuzkoa, Spain, 2006–2015.

Date (mo/y)	Age (y)	Sex	Comorbidities and predisposing factors	STSS	Invasive	Affected lung area	Fine	30-d mortality (days to death)	Biweekly influenza threshold (per 100,000)	Coinfection	Isolation site	*emm*	ST	Exotoxins genes
1/2006	61	Male	IST	-*	Yes	RUL (cavitary lesions)	V	-	< 80	-	Blood	1.0	28	*slo*, *speB*, *speC*, *smeZ*
04/2006	80	Male	COPD, AHT, RF, atrial fibrillation	-	-	RUL	IV	-	< 80	*Staphylococcus aureus*	BS	-	-	-
02/2007	55	Female	-	-	-	RLL+ RUL	II	-	355	Viral study not performed	BS	6.4	382	*slo*, *speB*, *speC*
05/2007	59	Male	COPD, AHT, DM	-	-	RLL	II	-	< 80	-	BS	6.0	382	*slo*,*speB*, *speC*
01/2008	63	Male	COPD, DM	-	-	RLL+ LLL	IV	-	296	Viral study not performed	BS	1.0	28	*slo*, *speA*, *speB*, *smeZ*
02/2008	44	Male	-	Yes	Yes	RLL+ LLL pleural effusion	V	-	265	Viral study not performed	Blood+ BS	1.0	28	*slo*, *speA*, *speB*, *smeZ*
02/2008	44	Female	-	Yes	Yes	LLL + pleural effusion	V	-	168	Viral study not performed	Blood+ BS	5.46	99	*slo*, *speB*, *speC*
02/2008	46	Male	Smoker, AHT, alcohol abuse	-	Yes	RUL	III	-	127	Viral study not performed	Blood+ BS	1.0	28	*slo*, *speA*, *speB*, *smeZ*
04/2008	44	Male	Cerebral palsy (nursing home)	-	-	LLL	III	-	< 80	-	BS	6.0	382	*slo*, *speB*, *speC*
06/2008	45	Male	Smoker, alcohol abuse	-	Yes	RLL+ LLL+ pleural effusion	V	-	< 80	-	Blood	1.0	28	*slo*, *speA*, *speB*, *smeZ*
07/2008	72	Male	COPD, DM, AHT, atrial fibrillation, stroke	-	Yes	RLL	V	-	< 80	-	Blood+ BS	87	62	*slo*, *speB*, *speC*, *smeZ*, *ssa*
10/2008	63	Female	AHT	-	-	LLL	ND	-	< 80	-	BS	4.0	39	*slo*, *speB*, *speC*, *smeZ*, *ssa*
05/2009	41	Male	-	-	-	RUL	I	-	< 80	-	BS	3.1	15	*slo*, *speA*, *speB*, *ssa*
06/2010	64	Male	COPD	-	-	RLL	IV	-	< 80	-	BS	12.0	36	*slo*, *speB*
04/2011	66	Male	AHT, IST, RF, HD (nosocomial)	-	-	RUL+RML+ RLL+ LLL	IV	Death (20 d)	< 80	*Pseudomonas aeruginosa*	BS	81	624	*slo*, *speB*
12/2011	63	Male	-	-	-	RLL	I	-	< 80	*Haemophilus influenzae*	BS	6.0	382	*slo*,*speB*, *speC*
02/2012	78	Male	AHT, HD, atrial fibrillation	-	-	RLL+ LLL	IV	-	327	Viral study not performed	BS	1.0	28	*slo*, *speA*, *speB*, *smeZ*
02/2012	39	Female	-	Yes	Yes	RLL+LLL	V	Death (<24 h)	229	-	Blood + BS	1.0	28	*slo*, *speA*, *speB*, *smeZ*
03/2012	54	Female	-	-	Yes	RLL	I	-	< 80	-	Blood	1.0	28	*slo*, *speA*, *speB*, *smeZ*
03/2012	67	Female	AHT, DM, IST, neoplastic disease	-	-	RUL (cavitary lesions)	V	-	< 80	-	BS	1.0	28	*slo*, *speA*, *speB*, *smeZ*
03/2012	90	Male	COPD, RF	Yes	Yes	RLL	V	Death (<24 h)	< 80	-	BS	6.0	382	*slo*, *speB*, *speC*
04/2012	46	Male	Smoker, alcohol abuse	Yes	Yes	RLL+ LLL+ pleural effusion	V	Death (<24 h)	< 80	-	Blood	1.0	28	*slo*, *speA*, *speB*, *smeZ*
11/2012	87	Male	AHT, DM	-	-	RLL+ LLL+ pleural effusion	V	-	< 80	-	BS	75.0	150	*slo*, *speB*, *speC*
03/2013	70	Female	IST	Yes	Yes	RLL+ RUL	V	Death (<24 h)	144	Influenza B	Blood + BS	1.0	28	*slo*, *speA*, *speB*, *smeZ*
08/2013	64	Female	AHT	-	-	LLL	III	-	< 80	*Haemophilus influenzae*	BS	75.0	150	*slo*, *speB*, *speC*
12/2013	74	Male	Smoker, AHT, HD,RF (nursing home)	-	Yes	RLL + pleural effusion	V	-	181	-	Blood	89.0	101	*slo*, *speB*
01/2014	59	Male	AHT	Yes	Yes	RLL	V	Death (3 d)	249	-	Blood	3.1	315	*slo*, *speA*, *speB*, *ssa*
01/2014	47	Male	AHT, Morbid obesity	-	Yes	LLL+ RML+ RLL pleural effusion	IV	-	249	Influenza H1N1pdm09	Blood + BS	1.0	28	*slo*, *speA*, *speB*, *smeZ*
01/2014	74	Male	AHT, DM	-	-	RUL+ RML	V	-	296	-	BS	3.1	15	*slo*, *speA*, *speB*, *ssa*
02/2014	86	Female	COPD, AHT, DM	-	Yes	RLL+ LLL	IV	Death (7 d)	< 80	-	Blood	1.0	28	*slo*, *speA*, *speB*, *smeZ*
03/2014	77	Male	COPD, DM, HD	-	Yes	LLL	V	-	< 80	-	BS	3.1	315	*slo*, *speA*, *speB*, *ssa*
03/2014	38	Female	-	Yes	Yes	LLL	V	Death (3 d)	< 80	-	BS	1.0	28	*slo*, *speA*, *speB*, *smeZ*
01/2015	25	Female	-	Yes	Yes	RLL + LLL pleural effusion	IV	-	318	-	Blood + BS	1.0	28	*slo*, *speB*, *smeZ*
01/2015	73	Male	IST	-	-	RLL pleural effusion (cavitary lesions)	IV	-	318	Influenza A H3	BS	3.1	15	*slo*, *speA*, *speB*, *ssa*
02/2015	37	Female	Pregnant	-	Yes	LLL + pleural effusion	III	-	484	Influenza A H3	Blood	3.39	315	*slo*, *speA*, *speB*, *ssa*
02/2015	32	Female	-	-	-	RUL + LUL (cavitary lesions)	I	-	292	-	BS	1.0	28	*slo*, *speB*, *smeZ*
02/2015	49	Male	Smoker	-	-	LLL	II	-	215	Influenza suspected. Viral study not performed	BS	89.0	101	*slo*, *speB*, *speC*
03/2015	44	Male	-	-	-	LLL	I	-	172	-	BS	1.0	28	*slo*, *speA*, *speB*, *smeZ*
03/2015	50	Female	-	Yes	Yes	RLL + pleural effusion	IV	-	122	Viral study not performed	Pleural fluid	77.0	63	*slo*, *speB*
12/2015	62	Male	IST	-	Yes	LLL	IV	-	< 80	-	Blood	4.0	39	*slo*, *speB*, *speC*, *smeZ*, *SSA ssa*

Abbreviations: STSS, streptococcal toxic shock syndrome; ST, sequence type; IST, immunosuppressive therapy; Smoker, heavy smoker; COPD, chronic obstructive pulmonary disease; AHT, arterial hypertension; RF, renal failure; BS, bronchial secretion; DM, diabetes mellitus; HD, heart disease (excluding hypertension); ICU, intensive care unit; RUL, right upper lobe; RML, right middle lobe; RLL, right lower lobe; LLL, left lower lobe; Fine at admission

*-: Negative

All but two cases included in the study were hospitalized. The age of patients ranged from 25 to 90 years with a median of 58.4 years and a mean of 60 years. Overall, 32.5% of cases occurred in patients older than 64 years (incidence 1.48 x100,000 elderly people). Nearly two-thirds (n = 25, 62.5%) of the patients were male.

All but 3 cases (1 nosocomial and 2 in nursing home residences) were community-acquired. The nosocomial pneumonia in a 66-year-old patient with a kidney transplant and multiple comorbidities, who died 20 days after admission, had abundant growth of *S*. *pyogenes* and *Pseudomonas aeruginosa* in bronchial secretions. This patient had also a cytomegalovirus infection.

Overall, in 20% of cases coinfections were detected, although viral coinfection was not investigated in some cases (Table 1). In addition to the patient coinfected with *P*. *aeruginosa* and cytomegalovirus, another 7 coinfections were: 2 with H3N2 Influenza A virus, 1 with H1N1pdm09 influenza A virus, 1 with influenza B virus, 2 with non-encapsulated *Haemophilus influenzae*, and 1 with *Staphylococcus aureus*.

### Clinical findings

In 21 patients (52.5%), *S*. *pyogenes* pneumonia presented as an invasive infection. In 17 of them, *S*. *pyogenes* was obtained from blood and in 1 from pleural fluid. The remaining 3 episodes were considered invasive infections even though the microorganism was only isolated in low respiratory secretions, as all had severe sepsis with renal failure, metabolic acidosis, hemodynamic instability, and elevated procalcitonin and C-reactive protein levels. Two of them developed STSS and died.

Twenty-seven patients had severe disease (Fine IV or V). Ten patients developed STSS. In 15 patients, pneumonia was multilobar. In 33 episodes (82.5%), the lower lobes were affected, 11 patients presented with pleural effusion, and cavitation was evidenced in 4 (Table 1).

### Comorbidities and predisposing factors

Notable underlying medical conditions were the presence of hypertension (37.5%), chronic obstructive pulmonary disease (COPD) (20%), and diabetes (20%) (Table 1). No comorbidities were detected in 13 patients (32.5%), although a woman without comorbidities was 35 weeks pregnant.

Various antibiotic regimens were used, the most commonly prescribed being a combination of a beta-lactam with clindamycin (n = 11) or with levofloxacin (n = 10), monotherapy with a betalactam (n = 10), monotherapy with levofloxacin (n = 7) or a combination of a beta-lactam with clindamycin and levofloxacin (n = 2). No relationship between the treatment received and mortality or clinical course was found.

### Mortality (case fatality)

The overall 30-day case fatality was 20% (8/40), which increased to 30.8% (4/13) in patients older than 64 years. Half of the patients with a fatal outcome (4/8), died within 24 hours after admission. Among lethal cases, there were 2 women aged 38 and 39 years without comorbidities or predisposing conditions.

In the multivariate logistic regression analysis, STSS was significantly associated with mortality (*p* = .004; odds ratio, 21.4; 95% CI, 2.7–170.7).

### Molecular Characterization of Isolates

**Fig 1:**
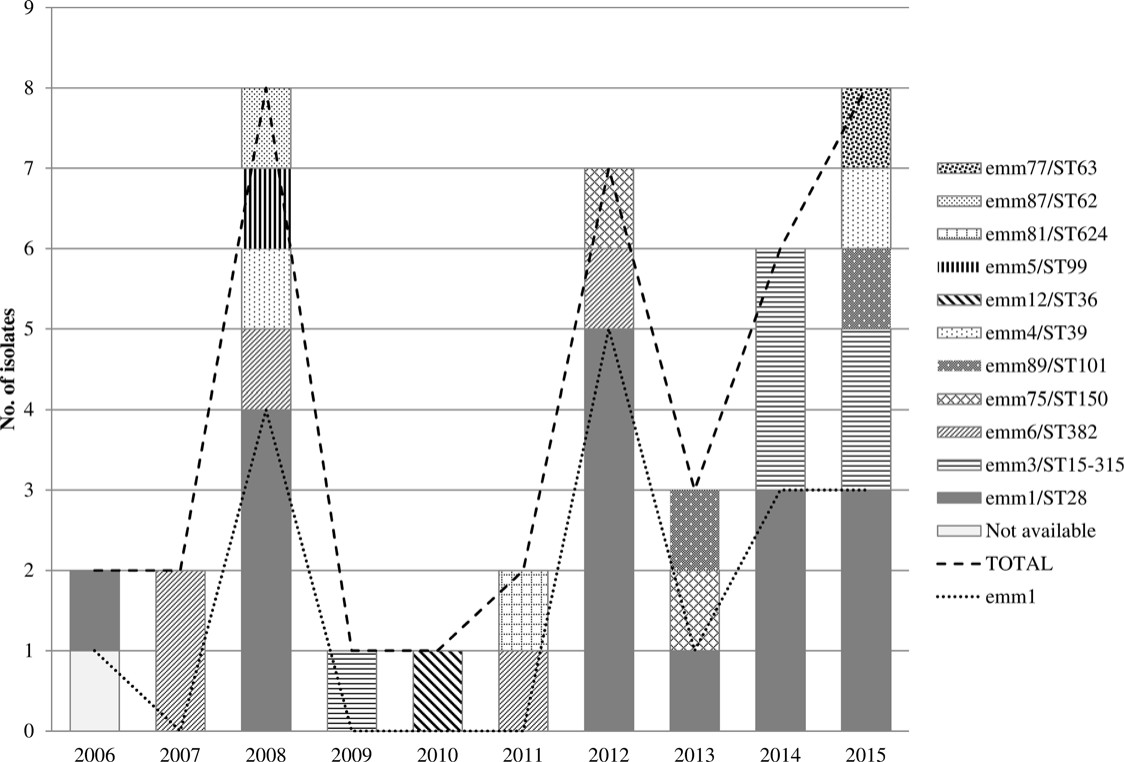
Annual distribution of adult *Streptococcus pyogenes* pneumonia episodes (n = 40) and involved clones. Gipuzkoa, Spain, 2006–2015.

All isolates except one were available for molecular characterization (39/40). Eleven clones were identified (Fig 1). The most prevalent clone was *emm*1/ST28 (43.6%, 17/39), followed by *emm*3/ST15-315 (15.4%, 6/39), and *emm*6/ST382 (12.8%, 5/39). *emm*3/ST15 and *emm*3/ST315 isolates belong to the same clonal complex (http://www.phyloviz.net/goeburst/) and in this article were considered as the same clone. A sudden increase in case number was observed in cycles of 3–4 years, coinciding with the highest *emm*1/ST28 pneumonia cases (Fig 1).

The *emm*1/ST28 clone was implicated in 62.5% (5/8) of fatal outcomes, in 73.3% (11/15) of patients with multilobar pneumonia and in 60% (6/10) of patients who developed STSS (Table 1).

The clones *emm*1/ST28 and *emm*3/ST15-ST315 were detected when seasonal influenza was above the epidemic threshold in 58.8% (10/17) and 66.7% (4/6) of times, respectively (Table 1).

### Antimicrobial Susceptibility

All isolates were susceptible to penicillin (MIC < 0.06 mg/L), and clindamycin (MIC < 0.25mg/L). Only 1 isolate, an *emm*12/ST36 strain, showed resistance to macrolides; it harbored the *mef* gene and expressed the M phenotype of resistance (erythromycin MIC = 1 mg/L). Five isolates (12.5%) showed low-level levofloxacin resistance (three isolates MIC = 2 mg/L and two MIC = 4 mg/L), and all were characterized as *emm*6/ST382 (*emm*6.0 n = 4 and *emm*6.4 n = 1), and harbored the Ser79/Ala mutation in the *parC* gene, but no mutation was detected associated with fluoroquinolone resistance in the *gyrA* gene.

### Exotoxins and Superantigen Profile

The streptococcal cysteine protease (*speB*) and the cytolytic toxin streptolysin O (*slo*) were present in all isolates. Overall, the most predominant superantigens genes among pneumonia isolates were *speA* (51.3%), *smeZ* (51.3%), *speC* (33.3%), and *ssa* (23.1%). A conservative superantigen profile was detected when the analysis was performed by clone. Isolates belonging to the *emm*1/ST28 clone harbored the *smeZ* (100%) and *speA* (82.4%). All *emm*3/ST15-315 isolates harbored the *speA* and *ssa* genes, and all *emm*6/ST382, *emm*75/ST150 and *emm*4/ST39 the *speC* gene (Table 1).

## Discussion

In the 21^st^ century, *S*. *pyogenes* pneumonia has been infrequently reported in detail. Since 2000, only 1 large series has been published describing the clinical presentation, prognosis and characteristics of the isolates [7]. Other publications have reported pneumonia as the clinical manifestation comprising the 7% to 11% of invasive *S*. *pyogenes* disease [6,16,17], but did not include specific details of these pneumonic episodes. The former articles, 1 outbreak in a military population [18], and some case reports [9–11], constitute the bulk of current information on this clinical entity. Therefore, is not surprising that *S*. *pyogenes* is not listed among CAP etiologies [19], or is only mentioned as a sporadic bacterial complication of influenza infection [4]. The incidence of most causes of CAP is not well defined. Apart from *S*. *pneumoniae* and *Legionella pneumophila*, *Mycoplasma pneumoniae* and *Chlamydia pneumoniae* are among persistently listed CAP etiologies [4,19]. The pneumonias caused by the latter two bacteria have a relative low fatality rate, and the incidence of pneumonia in adults is not too far from that obtained for *S*. *pyogenes* in the present series. In adult and children patients with CAP requiring hospitalization in U.S., Jain *et al*. found a similar presence of *C*. *pneumoniae* as *S*. *pyogenes* although a higher prevalence of *M*. *pneumoniae* [20,21]. Even so, some of these diagnoses were not specific enough as *C*. *pneumoniae* and *M*. *pneumoniae* were detected in nasopharyngeal or oropharyngeal swab by means of PCR assay. *M*. *pneumoniae* was found to be carried in the upper respiratory tract of a relatively high percentage of healthy, asymptomatic patients [22].

Annual average incidence of *S*. *pyogenes* pneumonia was of 1.14 episodes per 100,000 inhabitants (range 0.29–2.29). We assume it represents the minimum incidence as the study was a laboratory based surveillance study and some *S*. *pyogenes* pneumonia caseslikely were not included in the study. The main finding in the present series was that *S*. *pyogenes* pneumonia was a severe clinical entity with a continuous presence in the community. Most cases occurred in winter and early spring (December to April), as observed in other series [7,8], and mainly affected males, especially elderly or adults with comorbidities. The only source of *S*. *pyogenes* infections are human beings and the most frequent focus of infection is the oropharynx. The pathogen can be introduced into the lungs through haematogenous spread or inhalation. The development of pneumonia in some individuals and not in others may depend on both host defenses and the virulence of the microorganism. Among the 40 cases, most patients (26/40 65%) were adults with comorbidities or other important risk factors, although remarkably there were 13 previously healthy people. Six of these healthy people developed severe symptoms (Fine IV or V) and two died.

Among the known risk factors facilitating aspiration of oropharyngeal secretions (decreased consciousness, neurological disorders) surveyed in the study, alcoholism or neurological disorders were evidenced in only 4 patients. Increased age is another risk factor that not only facilitates aspiration of oropharyngeal secretions, but is also associated with a weakened immune response and the presence of more comorbidities. In the present series, half of the patients (n = 20) were older than 60 years. The comorbidities most frequently encountered (hypertension, chronic obstructive pulmonary disease, diabetes mellitus, heart disease, and renal failure) coincided with those commonly found in patients with pneumonia, independently of the causal pathogen [7,23,24]. Apart from mortality, a number of features evidenced the severity of *S*. *pyogenes* pneumonia episodes: in more than half of the patients, the pneumonia coursed as an invasive infection, in 18 (45%) patients more than 1 lobe or cavitation was involved, and Fine severity index score placed more than 65% of patients in the intermediate to high risk group.

The 30-day death rate was remarkably high (20%), which elevated to 33.3% among invasive cases and to 30.8% in patients older than 64 years. The fatality rate associated to invasive pneumonia was higher than that associated to other invasive *S*. *pyogenes* infections [6,7,16]. Mortality rates higher than 30% have been reported by other authors in pneumonic patients with *S*. *pyogenes* invasive infections [7,8]. In the present series half of the patients with a fatal outcome showed rapid onset, dying in less than 24 hours after admission despite the implementation of aggressive supportive care measures and appropriate antibiotic therapy, coinciding with that observed in reports of fulminant *S*. *pyogenes* pneumonia [8,9].

The 20% of patients presented with mixed infections, 4 of them (10%) with influenza virus. Most CAP occur in the cold months, coinciding with the circulation of respiratory viruses. The relationship between influenza and *S*. *pyogenes* pneumonia in the present series was lower than usually reported [8, 25–27]. In the present series only 4 influenza coinfection episodes were confirmed and 1 suspected; and, although 19 pneumonias occurred during periods with high circulation of influenza virus, the remaining 21 episodes occurred outside these periods. The single episode of coinfection with influenza A H1N1pdm09 was detected in 2014, and surprisingly during the course of the main influenza pandemic of 2009, only one *S*. *pyogenes* pneumonia case was detected but it was not related with influenza virus. Co-infection with influenza B virus was detected in an elderly woman who died, highlighting the potential morbidity and mortality associated with influenza B virus in the context of concurrent influenza infection [27,28].

All but 3 pneumonic cases were community acquired. There have been reports of clusters of *S*. *pyogenes* pneumonia within the same family [29]. However, no clustering with any relationship among episodes was observed in the present study. We found that the same clone (*emm*1/ST28) caused an increase of pneumonic episodes in 2012 but no relationship between these patients could be established. This *emm*1/ST28 clone was the predominant (43.6%) one, followed by *emm*3/ST15-315 (15.4%) and *emm*6/ST382 (12.8%) clones. These *emm* types were also predominant in the series of Muller *et al* [7]. In 1981–1997, Barnham *et al*. found that 9 out 17 analyzed pneumonia episodes were caused by an *emm*1 isolate [8], and the most recently detected *S*. *pyogenes* pneumonia outbreak among Marine Corps personnel was due to an *emm*3/ST15 strain [18].

The increase of *emm*1/ST28 pneumonia cases each 3–4 years, was perhaps the consequence of some kind of herd immunity after an initial spread of this clone among a susceptible population, although the reason of the dynamics of circulation of clones is unclear.

Although the *emm*1/ST28 clone in the present series was not associated with death with statistical significance, it was involved in 62.5% of fatal outcomes, including 2 young adults without predisposing factors. Similarly, Santagati *et al* recently reported 3 cases of fulminant hemorrhagic pneumonia associated with *emm*1/ST28 strains in previously healthy patients [9]. In the present series, this clone was also related to 11 out of 15 episodes with multilobar pneumonia and in 3 of 4 episodes with cavitary lesions. The virulent potential of *emm*1 isolates is well documented the world over, with a coincident resurgence of severe invasive infections since the late 1980s [30]. Clinical and epidemiological data analysis, whole-genome sequencing analysis of large bacterial collections, and infection models in nonhuman primates have demonstrated that *emm*1 strains recovered after 1988 are more virulent than those before 1988 due to acquisition of new genetic material [31].

As previously observed, a conservative superantigen profile by clone was frequently found [16]. For instance, almost all *emm*1/ST28 harbored the *speA* and *smeZ* superantigen genes. Overall, *speA* (51.3%), *smeZ* (51.3%) and *speC* (33.3%) were the most frequent superantigens genes found among pneumonia isolates.

Fortunately, all isolates were susceptible to clindamycin, which comprises a key antimicrobial for the treatment of severe *S*. *pyogenes* infections because it halts exotoxin production. Nonetheless, we detected a worryingly high rate of low level resistance to fluoroquinolones (12.8%) among strains associated with pneumonia, due to the involvement of isolates characterized as *emm*6/ST382, a clone which intrinsically harbors a mutation in the *parC* gene [32].

Although the representativeness of the study is limited to a specific geographic area, our findings suggest that today, as in the pre-antibiotic era, *S*. *pyogenes* pneumonia remains a very serious clinical entity, especially when it is associated with *emm*1 strains. *S*. *pyogenes* pneumonia is more common than is generally supposed and is often unrelated to influenza infection. Although it affects mainly debilitated people, it can be rapidly fatal even in previously healthy individuals.
